# Influence of the arterial input sampling location on the diagnostic accuracy of cardiovascular magnetic resonance stress myocardial perfusion quantification

**DOI:** 10.1186/s12968-021-00733-4

**Published:** 2021-03-29

**Authors:** Xenios Milidonis, Russell Franks, Torben Schneider, Javier Sánchez-González, Eva C. Sammut, Sven Plein, Amedeo Chiribiri

**Affiliations:** 1 School of Biomedical Engineering & Imaging Sciences, King’s College London, London, UK; 2 Philips Healthcare, Guilford, UK; 3 Philips Healthcare, Madrid, Spain; 4 Bristol Heart Institute and Translational Biomedical Research Centre, Faculty of Health Science, University of Bristol, Bristol, UK; 5 Leeds Institute of Cardiovascular and Metabolic Medicine, University of Leeds, Leeds, UK

**Keywords:** Cardiovascular magnetic resonance, Myocardial perfusion quantification, Myocardial blood flow, Myocardial perfusion reserve, Arterial input, Myocardial ischemia, Coronary artery disease

## Abstract

**Background:**

Quantification of myocardial blood flow (MBF) and myocardial perfusion reserve (MPR) by cardiovascular magnetic resonance (CMR) perfusion requires sampling of the arterial input function (AIF). While variation in the AIF sampling location is known to impact quantification by CMR and positron emission tomography (PET) perfusion, there is no evidence to support the use of a specific location based on their diagnostic accuracy in the detection of coronary artery disease (CAD). This study aimed to evaluate the accuracy of stress MBF and MPR for different AIF sampling locations for the detection of abnormal myocardial perfusion with expert visual assessment as the reference.

**Methods:**

Twenty-five patients with suspected or known CAD underwent vasodilator stress-rest perfusion with a dual-sequence technique at 3T. A low-resolution slice was acquired in 3-chamber view to allow AIF sampling at five different locations: left atrium (LA), basal left ventricle (bLV), mid left ventricle (mLV), apical left ventricle (aLV) and aortic root (AoR). MBF and MPR were estimated at the segmental level using Fermi function-constrained deconvolution. Segments were scored as having normal or abnormal perfusion by visual assessment and the diagnostic accuracy of stress MBF and MPR for each location was evaluated using receiver operating characteristic curve analysis.

**Results:**

In both normal (300 out of 400, 75 %) and abnormal segments, rest MBF, stress MBF and MPR were significantly different across AIF sampling locations (*p* < 0.001). Stress MBF for the AoR (normal: 2.42 (2.15–2.84) mL/g/min; abnormal: 1.71 (1.28–1.98) mL/g/min) had the highest diagnostic accuracy (sensitivity 80 %, specificity 85 %, area under the curve 0.90; *p* < 0.001 versus stress MBF for all other locations including bLV: normal: 2.78 (2.39–3.14) mL/g/min; abnormal: 2.22 (1.83–2.48) mL/g/min; sensitivity 91 %, specificity 63 %, area under the curve 0.81) and performed better than MPR for the LV locations (*p* < 0.01). MPR for the AoR (normal: 2.43 (1.95–3.14); abnormal: 1.58 (1.34–1.90)) was not superior to MPR for the bLV (normal: 2.59 (2.04–3.20); abnormal: 1.69 (1.36–2.14); *p* = 0.717).

**Conclusions:**

The AIF sampling location has a significant impact on MBF and MPR estimates by CMR perfusion, with AoR-based stress MBF comparing favorably to that for the current clinical reference bLV.

**Supplementary Information:**

The online version contains supplementary material available at 10.1186/s12968-021-00733-4.

## Background

First-pass cardiovascular magnetic resonance (CMR) perfusion imaging is a sensitive and accurate technique for the detection of coronary artery disease (CAD) [1–3]. While qualitative visual assessment of CMR perfusion datasets is the standard in clinical practice for guiding management of patients with suspected CAD [1, 3], it does not allow direct estimation of the severity of the disease and can be inaccurate when global reductions in myocardial blood flow (MBF) are present [4]. Recent studies have shown that full quantification of absolute MBF and myocardial perfusion reserve (MPR) by CMR can accurately identify surrogate markers of myocardial ischemia [5, 6], and has been validated against invasive fractional flow reserve (FFR) and positron emission tomography (PET) perfusion [7–9].

Central to MBF quantification is the arterial input function (AIF) which describes the contrast agent input to the myocardium [10]. It has been known since early theoretical developments of tracer-based flow measurement that accurate quantification requires the AIF to be sampled at the true myocardial input [11, 12]. However, imaging the relatively small coronary ostia is not a feasible option in CMR perfusion due to limitations of spatial resolution, which could result in severe partial volume effects exacerbated by cardiac and respiratory motion. Current perfusion quantification pipelines measure the AIF in the much larger basal left ventricular (LV) cavity instead, which offers the additional advantage of being imaged in the same slice as the myocardium and is usually free from myocardial trabeculation.

Variations in the AIF sampling location are known to impact rest and hyperemic MBF measurements by both PET and CMR perfusion quantification [13, 14]. An overestimation of absolute myocardial perfusion has potential to reduce the perceived ischemic burden and consequently affect the clinical interpretation of perfusion maps and decision-making in the clinic. Sampling the AIF in the aortic root (AoR) is feasible with contemporary dual-sequence implementations for perfusion imaging that decouple the AIF and myocardial signal acquisitions, and its closer proximity to the coronary ostia may favor this location over alternatives for perfusion quantification by CMR [14].

While we have previously described the impact of AIF sampling location on absolute MBF in a brief report [14], the impact on the diagnostic accuracy of MBF and MPR for ischemia detection is still unknown. In this study, using the retrospectively acquired subset of patients with known or suspected CAD included in the previous report, we provide a detailed assessment of the impact of the AIF sampling location on perfusion quantification in myocardium with and without inducible perfusion abnormalities. We also explore the diagnostic accuracy of stress MBF and MPR for different AIF sampling locations for the detection of abnormal myocardial perfusion.

## Methods

Twenty-five patients with suspected or known CAD referred for vasodilator stress perfusion CMR study as part of their clinical care were prospectively recruited. No patient had a history of aortic diseases. All patients gave written consent and the study was approved by the regional ethics committee (15/NS/0030). The study was conducted in agreement with the principles of the Declaration of Helsinki.

### CMR acquisition protocol

**Fig. 1:**
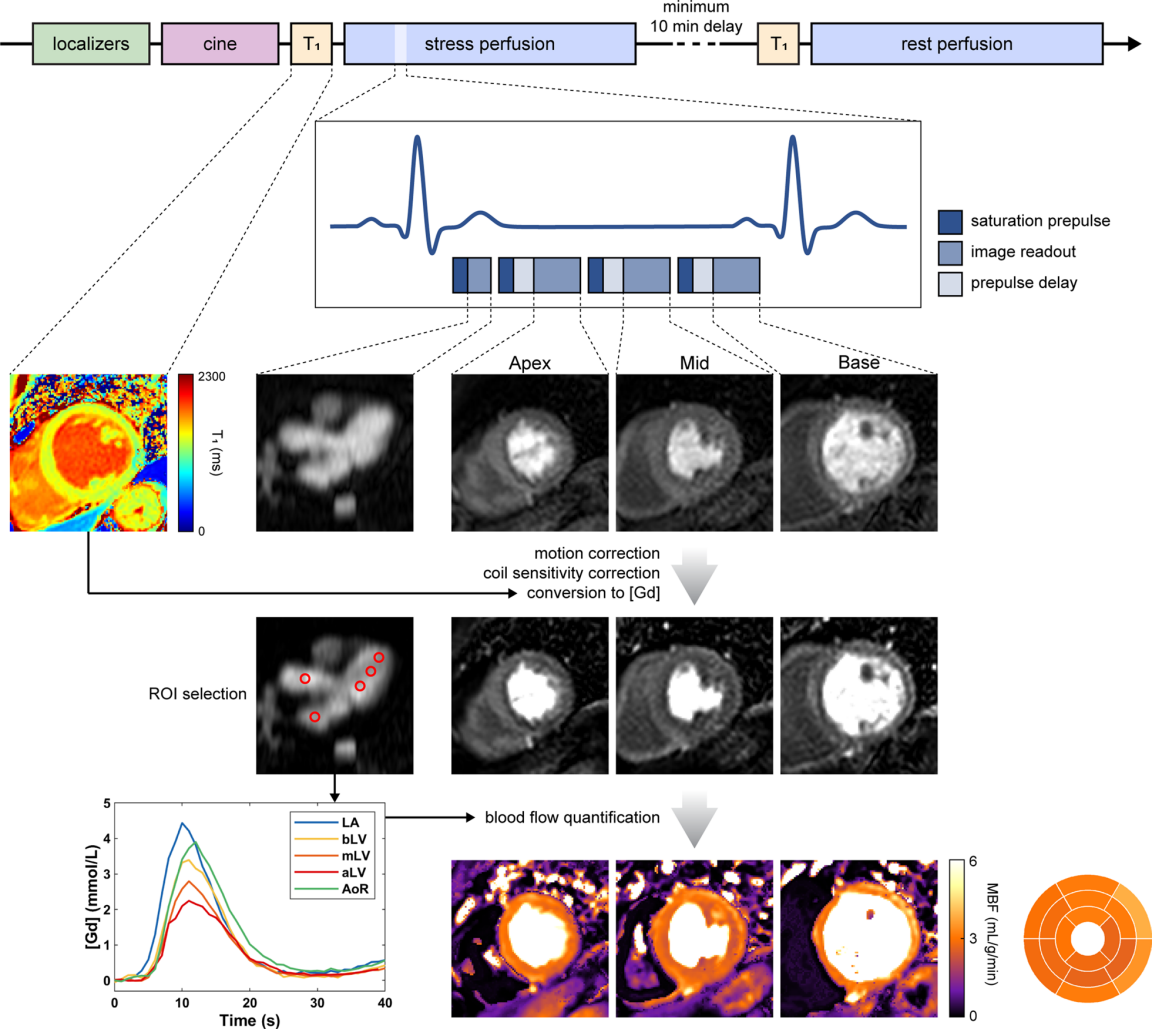
Overview of image acquisition and analysis protocol. Localizer and cine images are initially acquired to allow slice planning for perfusion imaging. Pre-contrast T_1_ mapping and first-pass perfusion imaging is performed at stress and rest with a minimum interval of 10 minutes. The low-resolution 3-chamber slice is acquired during systole and is followed by three high-resolution short-axis slices within each RR interval. Following normalization of images, the arterial input functions are manually derived from the low-resolution slice and myocardial blood flow (MBF) is quantified for each of the 5 locations at the pixel and segmental levels. The procedure shown is performed for both stress and rest perfusion imaging to estimate myocardial perfusion reserve. *aLV* apical left ventricular level, *AoR* aortic root, *bLV* basal left ventricular level, *LA* left atrium, *MBF* myocardial blood flow, *mLV* mid left ventricular level, *ROI* region of interest

Scanning was performed on a 3T CMR system (Achieva TX, Philips Healthcare, Best, The Netherlands) equipped with a 32-channel cardiac phased-array coil. Perfusion imaging was performed using an electrocardiogram (ECG)-triggered single-shot saturation recovery spoiled gradient echo dual-sequence implementation for myocardial perfusion [15], as shown in Fig. 1. A low-resolution slice was acquired in the 3-chamber (3Ch) orientation immediately after the R wave to image the passage of the contrast bolus through the left heart and ascending aorta. The low-resolution data were obtained using a fast readout with short saturation recovery time and typical parameters: TR 2.2 ms, TE 1.0 ms, saturation recovery time 23.5 ms, flip angle 15°, pixel bandwidth 1642 Hz, field of view 380 × 360 mm^2^, acquisition resolution 2.6 × 5.3 mm^2^, slice thickness 10 mm, SENSE acceleration factor 1.8. Three high-resolution short-axis slices through the basal, mid and apical LV cavity were acquired to assess myocardial perfusion (all sequence parameters as reported above except saturation recovery time 100 ms, acquisition resolution 2.6 × 2.6 mm^2^). Both low and high-resolution data were reconstructed to an in-plane resolution of 1.3 × 1.3 mm^2^. A bolus of 0.075 mmol/kg Gadobutrol (Gadovist®, Bayer AG, Leverkusen, Germany) was injected intravenously at 4 mL/s using an injector pump (Spectris Solaris, Medrad®, Bayer AG), followed by 25 mL of saline flush. Data were acquired for between 50 and 70 seconds after the injection of the contrast bolus. Two proton density images per slice were acquired at the beginning of each acquisition for estimating the coil sensitivity profile [16]. Prior to the acquisition of each perfusion scan, a modified Look-Locker inversion recovery (MOLLI) sequence (typical parameters: 5(3)3 sampling scheme, TR 3.1 ms, TE 1.2 ms, TI 157–4088 ms, flip angle 20°, pixel bandwidth 1085 Hz, field of view 350 × 350 mm^2^, acquisition resolution 2 × 2 mm^2^, slice thickness 10 mm) was used to obtain a T_1_ map for converting the signal intensity in perfusion data to gadolinium concentration [17]. Stress perfusion imaging was performed during adenosine-induced hyperemia (140 µg/kg/min for 3 minutes with a further 2 minutes at 175 µg/kg/min and a further 2 minutes at 210 µg/kg/min if an insufficient stress response had been achieved) [18]. Following a minimum delay of 10 minutes, a rest perfusion scan was acquired in accordance with published recommendations [19] (Fig. 1).

### Image analysis

Dynamic perfusion images first underwent motion correction and were corrected for coil sensitivity using the acquired proton density maps. Signal intensity (SI) was then converted to gadolinium concentration to account for the SI differences between the low- and high-resolution slices and correct for nonlinearity between SI and gadolinium concentration. All processing steps are described in detail in Additional file 1.

Five circular regions of interest (ROI) with a 10 mm diameter were manually placed over the motion-corrected low-resolution 3Ch slice to derive individual AIF curves for the following locations: left atrium (LA), basal left ventricular level (bLV), mid left ventricular level (mLV), apical left ventricular level (aLV) and aortic root (AoR). Other visible locations comprised the right ventricle and the descending aorta; these were excluded as the former includes the pulmonary circulation in its pathway and the latter is downstream of the coronary circulation and the blood stream has diverted into other aortic branches. The location of the five ROIs was selected in images exhibiting the highest gadolinium concentration over time within each region. The ROI in the AoR was placed as close as possible to the center of the sinus of Valsalva whilst avoiding the aortic valve. Where possible, the LV ROIs avoided the papillary muscles and trabeculation.

AIF curves were used to quantify absolute pixel-wise MBF for each sampling location using an automated pipeline employing Fermi function-constrained deconvolution [20]. The quantification pipeline first interpolates the images to a fixed temporal resolution [21, 22]. Each AIF is examined to automatically detect the time when the contrast first enters the region (upslope start), as well as the time separating the contrast first-pass and second-pass. For the detection of the upslope start time the triangle method described by Goldstein et al. is used [23]. The time separating the contrast first-pass and second-pass is identified by first detecting the corresponding peaks based on the first derivative of the curve and then detecting the minimum between the two peaks. Dynamics before and after these two time points are discarded, while the AIF upslope start is also used to temporally crop the tissue enhancement curves. A minimum time window of 20 seconds is used for deconvolution; if the cropped time window is less than this threshold then the window is extended at washout to include 20 seconds. Each tissue enhancement curve is examined to detect the time delay (time between the AIF upslope start and contrast arrival in the myocardium), which is then modelled as a unit step function in the Fermi model [20]. The fitting is repeated for 3 seconds before and after the estimated arrival time and the time providing the smallest residuals is selected [24]. Quantification is only performed for pixels within a 12 × 12 cm^2^ bounding box centered in the LV to reduce computation time.

The pixel-wise map for each short-axis slice is interpolated into a polar grid with 60 angular positions and 10 transmural layers, as described previously [25, 26], to allow assessment of perfusion at the segmental level. First, the endocardial and epicardial contours and the superior RV insertion point are manually selected on the motion-corrected short-axis images before quantification using a custom-written MATLAB tool (Mathworks, Natick, Massachusetts, USA). The contours are then used after quantification to split the myocardium in perfusion maps into 16 segments according to the American Heart Association (AHA) model [27]. MPR, defined as the stress-to-rest ratio of MBF, was measured by dividing MBF in corresponding segments. ROI placement was performed using ImageJ (version 1.51j8; Rasband, W.S., National Institutes of Health, Bethesda, Maryland, USA) by a cardiologist experienced in CMR. For all other analyses, automated or semi-automated MATLAB (version 2019b; MathWorks) routines developed in house were used.

The diagnostic accuracy of segmental stress MBF and MPR based on the different AIF sampling locations for detection of myocardial ischemia was assessed against expert visual assessment of first-pass perfusion images. Visual assessment was performed by two independent experts blinded to all other data (cvi42, version 5.6.4; Circle Cardiovascular Imaging Inc, Calgary, Alberta, Canada). An inducible perfusion abnormality was defined as a delayed wash-in of the contrast agent in a segment compared with non-ischemic myocardium lasting ≥ 5 dynamics that was not seen at rest and not related to obvious respiratory, motion or dark rim artefact [28]. The presence (‘abnormal’ segments) or absence (‘normal’ segments) of a perfusion abnormality was assigned to the appropriate segment on an AHA 16-segment model [27].

### Statistical analysis

The normality of data was assessed using the Shapiro-Wilk test. Patient characteristics, segmental MBF and MPR were not normally distributed and were summarized as median (interquartile range). Friedman’s 2-way analysis of variance by ranks was used to perform comparisons of measurements for the five AIF locations with post hoc pairwise comparisons using Dunn’s tests with Bonferroni correction. Linear regression analysis was used to assess the relationship between measurements for the clinical reference bLV and other AIF sampling locations. The agreement between locations was examined using Bland-Altman analysis. The diagnostic accuracy of stress MBF and MPR as compared to expert visual assessment was evaluated using receiver operating characteristic (ROC) curves for each of the five AIF sampling locations. Optimal cut-off values were determined using the Youden index and the area under the ROC curves was compared using the DeLong method with Bonferroni correction. The intraclass correlation coefficient was calculated to determine the need to adjust analyses for intra-patient clustering of measurements, as previously described [7, 29]. All tests were 2-tailed and statistical significance was set at *p* < 0.05. Statistical analysis was performed in SPSS® (version 25.0; Statistical Package for the Social Sciences, International Business Machines, Inc., Armonk, New York, USA) and R software (version 3.4.0; R Foundation for Statistical Computing, Vienna, Austria).

## Results

### Study population

Table 1 summarizes baseline characteristics of the study cohort (*n* = 25). The mean age of patients was 68 (52–75) years and 14 patients (56 %) were men. A total of 400 myocardial segments were available for analysis and were classified into normal (300 (75 %)) and abnormal (100 (25 %)) based on expert visual assessment of first-pass perfusion images. Abnormal segments were reported in 12 patients (48 %).

**Table 1:** Patient characteristics

Characteristic	*n* = 25
Male gender, *n* (%)	14 (56)
Age, years	68 (52–75)
Diabetes mellitus, *n* (%)	5 (20)
Dyslipidaemia, *n* (%)	15 (60)
Hypertension, *n* (%)	10 (40)
Previous revascularisation, *n* (%)	5 (20)
Never smoked, *n* (%)	12 (48)
Resting heart rate, beats/min	71 (64–79)
Stress heart rate, beats/min	92 (75–100)
LVEDVI, mL/m^2^	91 (69–102)
LVESVI, mL/m^2^	33 (27–57)
LVEF, %	59 (48–66)
Indexed LV mass, g/m^2^	46 (39–54)

Data expressed as median (interquartile range) or *n* (%). *LV* left ventricular, *LVEF* left ventricular ejection fraction, *LVEDVI* left ventricular end diastolic volume indexed, *LVESVI* left ventricular end systolic volume indexed

### MBF and MPR

In normal segments, rest MBF ranged from 0.95 (0.80–1.22) mL/g/min for the LA to 1.23 (0.96–1.52) mL/g/min for the aLV (*p* < 0.001). Measurements for all pairs of locations except the LA-AoR and mLV-aLV differed significantly (*p* ≤ 0.001). In abnormal segments, rest MBF ranged from 0.96 (0.85–1.21) mL/g/min for the AoR to 1.25 (1.02–1.50) mL/g/min for the aLV (*p* < 0.001). Measurements for the AoR differed significantly from all LV locations (*p* < 0.001).

**Fig. 2:**
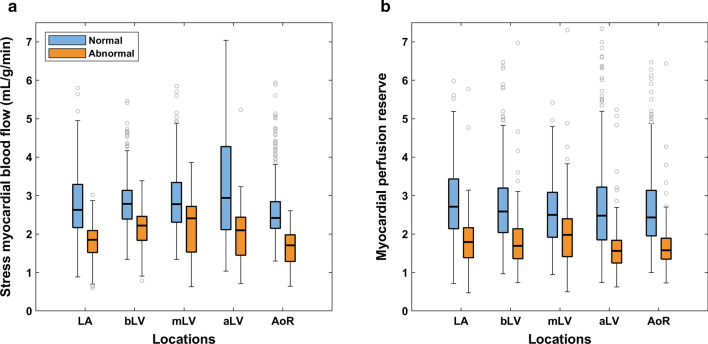
Segmental stress myocardial blood flow (**a**) and myocardial perfusion reserve (**b**) in normal and abnormal segments for five different arterial input sampling locations. The outliers are shown as gray circles. Pairwise comparisons are given in Additional file 1: Table S1. *aLV* apical left ventricular level, *AoR* aortic root, *bLV* basal left ventricular level, *LA* left atrium, *mLV* mid left ventricular level

In normal segments, stress MBF ranged from 2.42 (2.15–2.84) mL/g/min for the AoR to 2.94 (2.11–4.28) mL/g/min for the aLV (*p* < 0.001). Stress MBF for the AoR differed significantly from all LV locations (*p* < 0.001; Fig. 2a). In abnormal segments, stress MBF ranged from 1.71 (1.28–1.98) mL/g/min for the AoR to 2.41 (1.53–2.72) mL/g/min for the mLV (*p* < 0.001). Measurements for the bLV differed significantly with those for the AoR and the LA (*p* < 0.001).

In normal segments, MPR ranged from 2.43 (1.95–3.14) for the AoR to 2.71 (2.14–3.43) for the LA (*p* < 0.001), while in abnormal segments, MPR ranged from 1.56 (1.24–1.84) for the aLV to 1.98 (1.41–2.40) for the mLV (*p* < 0.001; Fig. 2b). MPR was similar between the bLV and AoR for normal (2.59 (2.04–3.20) versus 2.43 (1.95–3.14) respectively, *p* = 1.00) and abnormal (1.69 (1.36–2.14) versus 1.58 (1.34–1.90) respectively, *p* = 0.226) segments. *p*-values for all pairwise comparisons can be found in Additional file 1: Table S1.

**Fig. 3:**
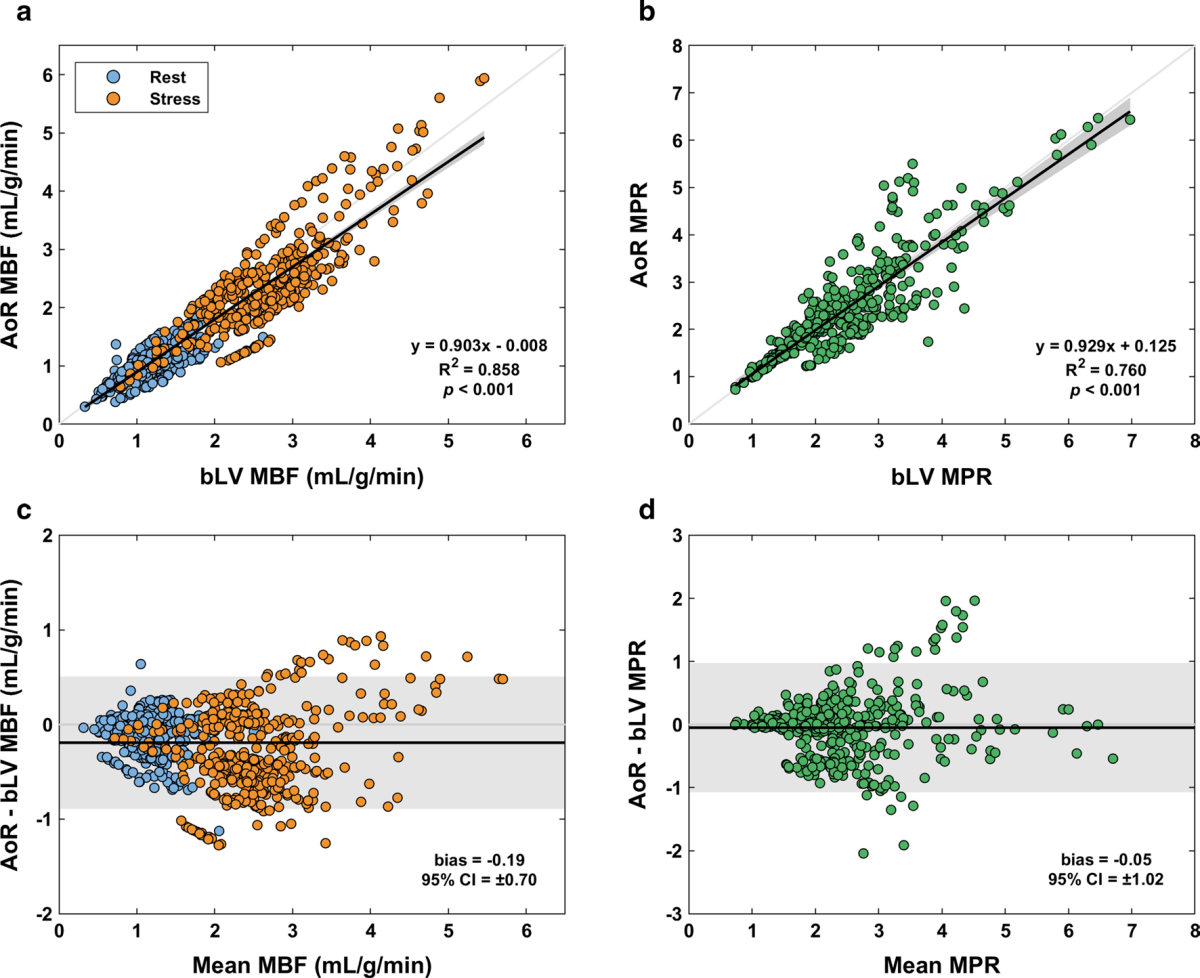
Linear regression for myocardial blood flow (MBF) (**a**) and myocardial perfusion reserve (MPR) (**b**) measurements between the aortic root (AoR) and the clinical reference basal left ventricle (bLV). Corresponding Bland-Altman plots are also shown (**c** and **d** respectively). The shaded areas show the 95 % confidence intervals (CI)

Table 2 summarizes the results of linear regression and Bland-Altman analysis. There was a significant relationship between segmental MBF for the bLV and other locations (*p* < 0.001), with the variation in AoR-based MBF explained best by the model (R^2^ = 0.858; Fig. 3a). Results were similar for MPR, for which a significant relationship was found between bLV and other locations (*p* < 0.001) and AoR-based MPR provided the best fit (R^2^ = 0.760; Fig. 3b). However, AIF sampling in the AoR underestimated both MBF and MPR compared to bLV (bias − 0.193 mL/g/min and − 0.051 respectively; Fig. 3c–d).

**Table 2:** Linear regression and Bland-Altman analysis of segmental perfusion

AIF sampling location	Slope (95 % CI)	R^2^	*p*-value	Bias (95 % CI)
MBF				
Left atrium	0.988 (0.959–1.017)	0.850	< 0.001	− 0.14 (0.76)
Mid-left ventricle	0.985 (0.955–1.015)	0.836	< 0.001	0.07 (0.80)
Apical left ventricle	1.102 (1.035–1.169)	0.567	< 0.001	0.21 (1.78)
Aortic root	0.903 (0.877–0.928)	0.858	< 0.001	− 0.19 (0.70)
MPR				
Left atrium	0.842 (0.781–0.902)	0.654	< 0.001	0.09 (1.23)
Mid-left ventricle	0.761 (0.712–0.811)	0.693	< 0.001	− 0.08 (1.09)
Apical left ventricle	0.578 (0.456-0.700)	0.177	< 0.001	− 0.01 (2.54)
Aortic root	0.929 (0.878–0.981)	0.760	< 0.001	− 0.05 (1.02)

Comparisons for each AIF sampling location were performed against the clinical reference basal left ventricle (bLV). The bias (mean difference) for MBF is measured in units of mL/g/min. Positive bias indicates measurement overestimation compared to bLV. *AIF* arterial input function, *CI* confidence interval, *MBF* myocardial blood flow, *MPR* myocardial perfusion reserve

### Diagnostic accuracy versus visual assessment

**Fig. 4:**
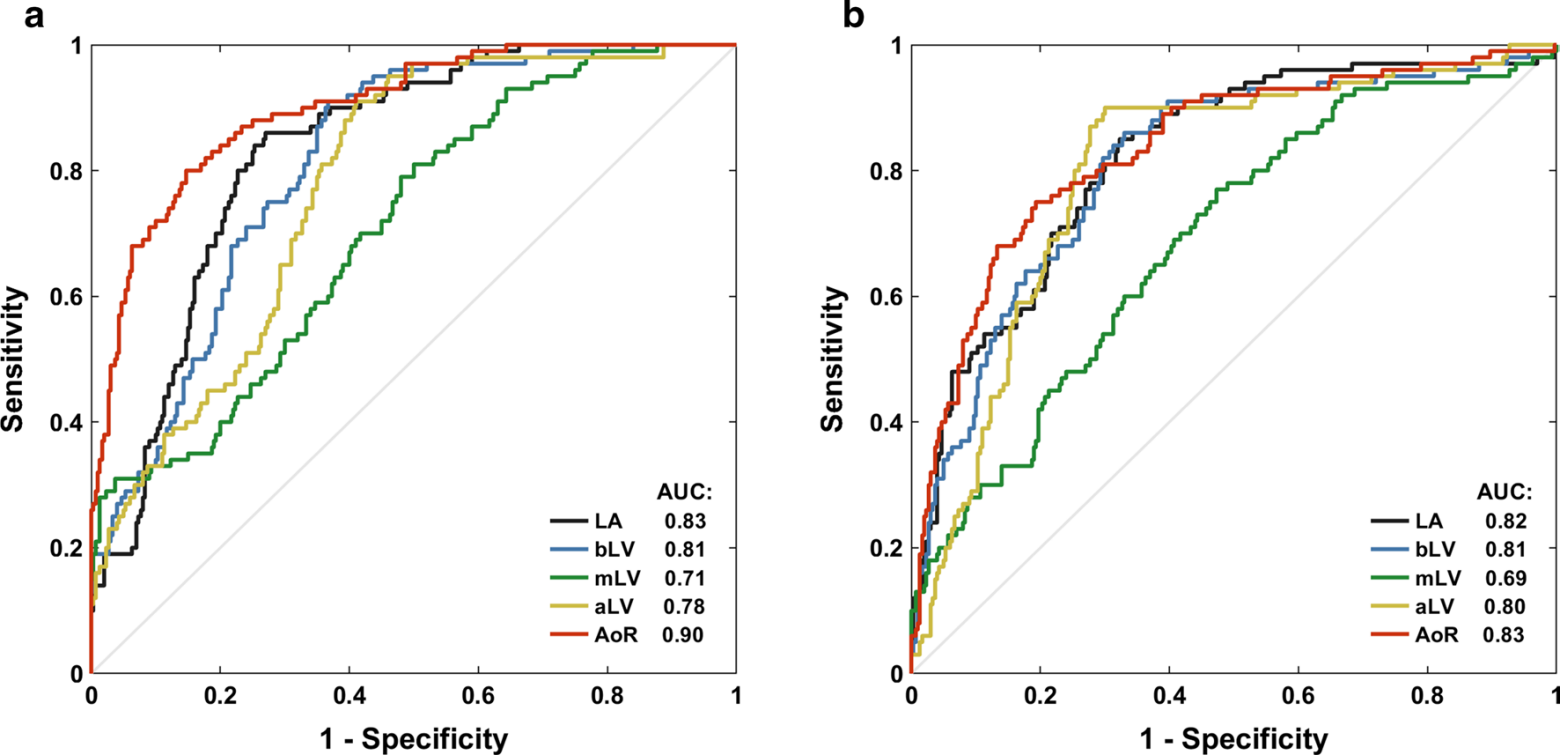
Receiver operating characteristic (ROC) curves for detection of abnormal myocardial perfusion by stress myocardial blood flow (**a**) and myocardial perfusion reserve (**b**) for five arterial input sampling locations. *aLV* apical left ventricular level, *AoR* aortic root, *bLV* basal left ventricular level, *LA* left atrium, *mLV* mid left ventricular level

Figure 4 shows ROC curves for all AIF sampling locations and Table 3 summarizes their diagnostic performance. Stress MBF for the AoR was the most accurate predictor of segments with abnormal perfusion with sensitivity 80 %, specificity 85 % and AUC 0.90 [95 % confidence interval (CI): 0.87–0.93] (*p* < 0.001; *p* < 0.001 versus stress MBF for all other locations). For the bLV, a high sensitivity but a moderate specificity was measured (91 % and 63 % respectively; AUC 0.81 [95 % CI: 0.77–0.85], *p* < 0.001). Consequently, the optimal MBF cut-off to distinguish between normal and abnormal myocardium was found to be considerably higher for the bLV than for the AoR (2.63 versus 2.02 mL/g/min).

**Table 3:** ROC analysis for detection of abnormal segments by stress MBF and MPR

AIF sampling location	AUC (95 % CI)	Sensitivity, %	Specificity, %	Optimal cut-off	*p*-value
Stress MBF					
Left atrium	0.83 (0.79–0.87)	86	73	2.24	< 0.001
Basal left ventricle	0.81 (0.77–0.85)	91	63	2.63	< 0.001
Mid-left ventricle	0.71 (0.66–0.75)	79	52	2.75	< 0.001
Apical left ventricle	0.78 (0.73–0.82)	91	59	2.65	< 0.001
Aortic root	0.90 (0.87–0.93)	80	85	2.02	< 0.001
MPR					
Left atrium	0.82 (0.78–0.87)	85	68	2.27	< 0.001
Basal left ventricle	0.81 (0.77–0.85)	86	67	2.25	< 0.001
Mid-left ventricle	0.69 (0.64–0.73)	77	53	2.42	< 0.001
Apical left ventricle	0.80 (0.76–0.84)	90	70	2.03	< 0.001
Aortic root	0.83 (0.79–0.87)	75	81	1.88	< 0.001

Results based on 300 normal and 100 abnormal segments as diagnosed visually. The optimal cut-off indicates the value below which the segment is classified as positive (inclusive). The cut-off for stress MBF is measured in units of mL/g/min

*AUC* area under the curve, *AIF* arterial input function, *CI* confidence interval, *MBF* myocardial blood flow, *MPR* myocardial perfusion reserve

MPR for the AoR detected inducible perfusion abnormalities with sensitivity 75 %, specificity 81 % and AUC 0.83 [95 % CI: 0.79–0.87] (*p* < 0.001; Fig. 4; Table 3). The diagnostic performance of bLV and AoR MPR was not significantly different (*p* = 0.717), but both were better than the worst-performing mLV (*p* < 0.001). When stress MBF and MPR ROC curves were compared, stress MBF for the AoR performed better than MPR for all LV locations (*p* < 0.01), however, stress MBF for the bLV was not superior to MPR for the same location or AoR (*p* = 1.000). In concordance with stress MBF, the optimal MPR cut-off to distinguish between normal and abnormal myocardium was larger for the bLV than for the AoR (2.25 versus 1.88 respectively). *p*-values for all pairwise comparisons can be found in Additional file 1: Tables S2, S3.

**Fig. 5:**
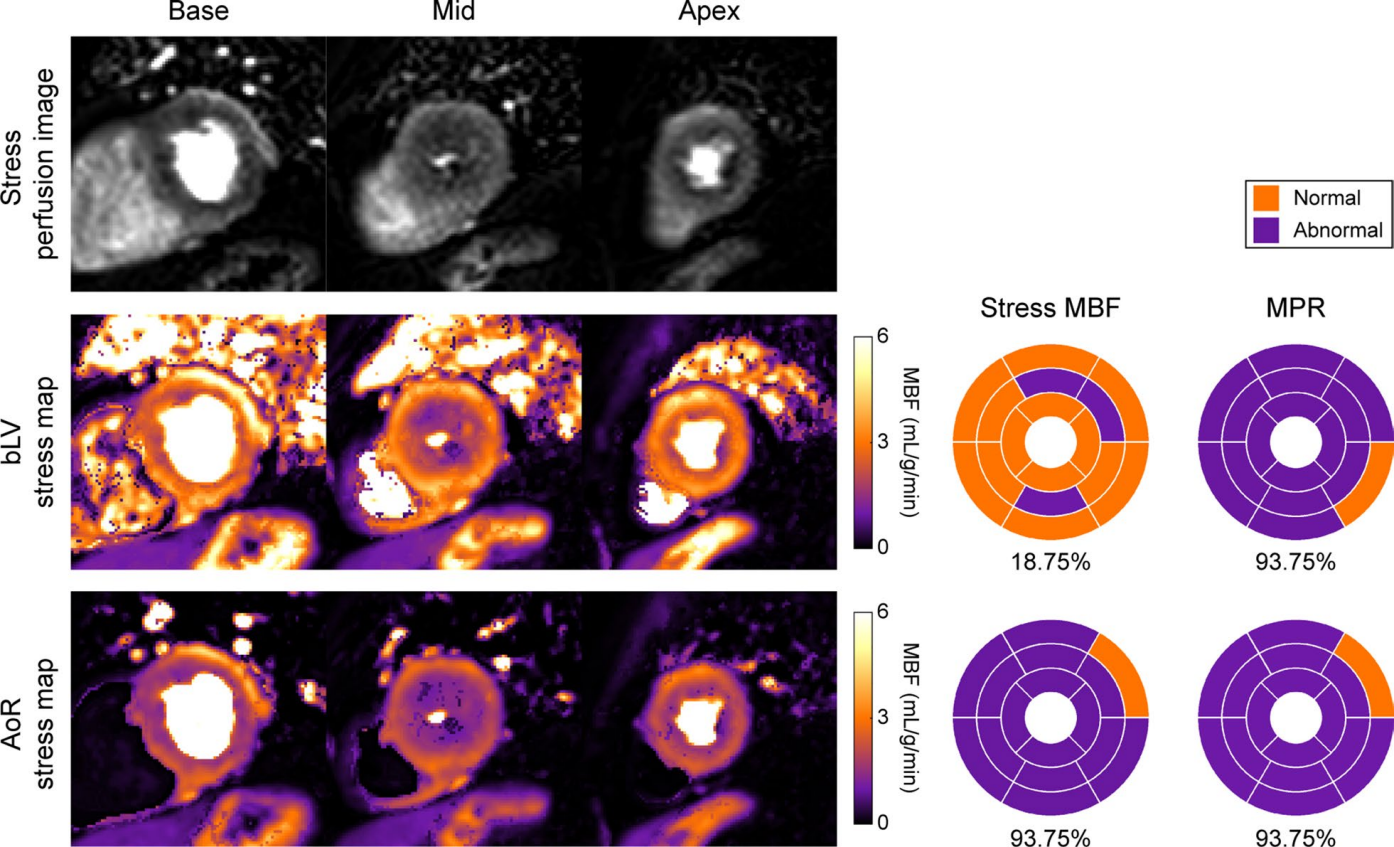
Impact of the arterial input function (AIF) sampling location on ischemic burden and clinical interpretation. Example stress perfusion images (top), pixel-wise MBF maps (bottom-left) and corresponding 16-segment bullseye plots (bottom-right) of myocardial blood flow (MBF) and myocardial perfusion reserve (MPR) demonstrating the impact on ischemic burden in a patient with widespread ischemia for the clinical reference basal left ventricle (bLV) and the aortic root (AoR). The mean stress MBF is 2.38 mL/g/min and 1.65 mL/g/min for the bLV and AoR respectively, and the mean MPR is 1.36 and 1.44 for the bLV and AoR respectively. The percent ischemic burden (given below each plot) was measured using the same AoR-derived cut-off values (stress MBF ≤ 2.02 mL/g/min and MPR ≤ 1.88), though a corresponding effect is observed using values for alternative locations

Figure 5 demonstrates the impact of the AIF sampling location on measured ischemic burden and clinical interpretation for a patient with widespread perfusion abnormalities when fixed cut-off values are used. The ischemic burden derived for stress MBF is considerably different for the bLV and the AoR despite the use of identical imaging data, whereas it remains similar for MPR.

## Discussion

This study evaluated the impact of the AIF sampling location on perfusion quantification in myocardium with normal and abnormal perfusion and supports the following main findings. First, perfusion quantification is impacted by the AIF sampling location when myocardial perfusion is both normal and abnormal. Second, MPR is less affected by variation in AIF sampling location than absolute stress MBF. Finally, variation of the AIF sampling location leads to different estimates of diagnostic accuracy for detection of abnormal myocardial perfusion, as well as different optimal MBF and MPR cut-off values. These findings have important implications for the interpretation of CMR perfusion data and encourage standardization of quantitative analysis to ensure reproducibility of perfusion measurements.

Quantification of perfusion is based on the indicator-dilution theory that relates the amount of contrast agent inflow with outflow [11, 12]. The principle postulates that all contrast agent molecules measured at the input location flow across the myocardial capillary bed and reach the output location, and therefore the principle assumes a single-input single-output system. However, current quantification pipelines violate this assumption as, owing to limitations of spatial resolution, the AIF cannot be sampled from the true input to the myocardium (the coronary arteries). Instead, the AIF is sampled in the bLV which is conveniently imaged in the same slice as the myocardium.

Due to the effects of ongoing dilution we would expect a gradual reduction in measured peak gadolinium concentration across the AIF sampling locations as the contrast bolus travels from the LA to the AoR. However, we found that AIF locations within the LV cavity lead to lower measured gadolinium concentrations and thus higher measured MBF than in the LA or AoR in both normal and abnormal myocardium. This is consistent with the findings of Vasquez et al. which explored a similar question in PET perfusion quantification, an imaging modality with a similar spatial resolution to the low-resolution AIF slice used in our study [13]. Similar findings on quantitative CMR perfusion were first described by Franks et al. [14], while a more recent study reported a significant difference on semi-quantitative AIF indices between LV and alternative locations [30]. We suggest the reason for this finding is likely multifactorial. First, the AIF slice is acquired in systole and with low resolution and therefore the LV locations are more prone to motion artefacts and partial volume effects due to LV contractility and trabeculation. This can further explain the overall increase in MBF from the base to the apex of the heart. Second, consistent with 3D phase contrast velocity imaging [31, 32], contrast within the LV may not be homogeneously mixed with blood suggesting that contrast enhancement in the cavity is also likely non-homogenous and could result in lower peak enhancement and subsequent over-estimation of myocardial blood flow. It is important to note that such an effect cannot be accounted for by coil sensitivity correction or conversion of SI to gadolinium concentration, processing steps that were applied in this study.

Our findings demonstrate that while stress MBF and MPR for all considered AIF sampling locations are able to distinguish between myocardium with normal and abnormal perfusion, perfusion measurements for the two groups are different depending upon the AIF location used. It is important to note that the diagnostic performance of the LV locations as assessed by ROC-AUC does not correlate with their anatomical location, as each may be impacted to a different extent by aforementioned factors. For example, the interquartile range in mLV-based stress MBF and MPR in abnormal segments is considerably larger than the corresponding range for bLV, leading to significantly lower diagnostic performance despite the close proximity of the two locations. AoR-based stress MBF and MPR were the strongest predictors of myocardial perfusion status. Furthermore, AoR-stress MBF was superior to both stress MBF and MPR for all other locations. This finding is consistent with the indicator-dilution theory from which we expect that the optimal AIF sampling location is the one closest to the true input to the myocardium [11, 33]. Automated detection of the AoR and derivation of an AIF with advanced machine learning techniques for use in inline quantification pipelines should be feasible, even in the case of aortic valve disease, as long as the low-resolution slice is adequately positioned and motion-corrected.

Although differences in MPR across locations were present in segments with both normal and abnormal perfusion, the difference was less pronounced than in stress MBF and indeed on pairwise comparison there was no significant difference between the MPR from AoR and bLV locations. This is expected since MPR normalizes stress MBF with the equivalently impacted rest MBF and thus has a decreased dependency on the AIF sampling location.

Rather than the binary presence of ischemia, it is ischemic burden that is considered the strongest predictor of patient prognosis [34–36]. In this study we have demonstrated that optimal stress MBF and MPR thresholds for identifying ischemia depend on the AIF sampling location and are not transferable between locations without a profound effect on the ischemic burden estimation and therefore on the prediction of patient risk. We therefore propose using thresholds of ischemia specific to the AIF sampling location.

### Limitations

One important limitation of our study is that the diagnostic accuracy of stress MBF and MPR was measured against expert visual assessment of perfusion images. Enrolled patients were referred to our tertiary center for a stress perfusion CMR study as part of their clinical care. As such, access to an optimal functional reference standard such as invasive physiology or PET data were not available. Although not an optimal reference, visual assessment of dynamic contrast-enhanced CMR perfusion studies has been validated to detect myocardial ischemia against FFR and indeed can be safely used to guide management of patients with suspected CAD [3]. While we have suggested a superiority of the AoR for detection of abnormal myocardial perfusion, we cannot suggest from this work that this would improve the clinical diagnosis of CAD and further validation against a reference standard is needed.

## Conclusions

AIF sampling location has a significant impact on myocardial perfusion estimates by CMR and can potentially impact clinical interpretation, particularly when absolute stress MBF values are used for diagnosis and risk stratification. MPR is less sensitive to the AIF sampling location. Stress MBF based on AIF sampling in the AoR leads to more accurate detection of inducible perfusion abnormalities and could be favored over alternative locations. This is supported by the indicator-dilution theory and is made possible by contemporary dual-sequence techniques for CMR perfusion that allow decoupling of the AIF and myocardial sampling locations. Future validation studies against a reference method, such as invasive FFR or PET perfusion, are needed to establish the superiority of the AoR for sampling the AIF required for perfusion quantification.

## Supplementary Information

**Additional file 1.** Additional tables.

## Data Availability

The datasets used and analyzed during the current study are available from the corresponding author upon reasonable request.

## Abbreviations

3Ch: 3-chamber
AHA: American Heart Association
AIF: Arterial input function
aLV: Apical left ventricular level
AoR: Aortic root
AUC: Area under the curve
bLV: Basal left ventricular level
CAD: Coronary artery disease
CI: Confidence interval.
CMR: Cardiovascular magnetic resonance
FFR: Fractional flow reserve
LA: Left atrium
LV: Left ventricle/left ventricular
LVEDVI: Left ventricular end-diastolic volume index.
LVEF: Left ventricular ejection fraction.
LVESVI: Left ventricular end-systolic volume index.
MBF: Myocardial blood flow
mLV: Mid left ventricular level
MOLLI: Modified Look-Locker inversion recovery
MPR: Myocardial perfusion reserve
PET: Positron emission tomography
ROC: Receiver operating characteristic
ROI: Region of interest
RV: Right ventricle
SI: Signal intensity
