# Supplementary material for: Influence of the arterial input sampling location on the diagnostic accuracy of cardiovascular magnetic resonance stress myocardial perfusion quantification
Source: J Cardiovasc Magn Reson. 2021 Mar 29;23:35. doi: 10.1186/s12968-021-00733-4 (PMC8006361; doi:10.1186/s12968-021-00733-4)
Supplement: Supplementary file 1 — Additional file 1. Additional tables. [file 12968_2021_733_MOESM1_ESM.docx]

**Additional file 1**

**Influence of the arterial input sampling location on the diagnostic accuracy of myocardial perfusion quantification by cardiovascular magnetic resonance**

Analyses described below were performed using automated or semi-automated tools developed in MATLAB (version 2019b; MathWorks^®^, Natick, Massachusetts, USA).

**Motion correction**

Dynamic perfusion images were motion-corrected using a rigid registration algorithm. First, the algorithm automatically detects the location of the left ventricle (LV) with a process similar to the validated approach described by Jacobs et al. (1). For this purpose, the maximum and mean intensity projections, their difference image, as well as the pixel-wise standard deviation of the basal left ventricular slice are obtained. The algorithm then looks in random patches in either of these images and generates a mask of the bright regions using an optimal thresholding method (2). Convergence is reached when both the LV and the right ventricle (RV) are detected based on pre-defined criteria of ventricular size and Euclidean distance between the ventricles. The signal intensity-time curve in both ventricles is sampled and compared to select the one with the latest peak enhancement, corresponding to the LV. Motion correction is then performed by registering each dynamic image on the left ventricular peak enhancement image, based on a 12x12 cm^2^ bounding box centered in the ventricle to ignore information from surrounding image regions. The regular step gradient descent optimizer is used for registration (3), while Mattes mutual information was used as the registration metric which handles dynamic changes in intensity better than the standard mean square error (4). Three iterations of the registration across all dynamics are used. Images may then be visually inspected and manually motion corrected, where deemed necessary. The motion correction algorithm was initially developed for use on short-axis images but was also applied on 3-chamber view images by adapting the criteria for convergence.

**Coil sensitivity correction**

Motion-corrected low-resolution 3-chamber view images and high-resolution short-axis images were normalized for B_1_ inhomogeneity due to the receive coil’s sensitivity (5). For this purpose, the pixel-wise mean of the two proton density (PD) images per slice is obtained and *k*-means clustering is used to select pixels corresponding to the visible structures and ignore low-density tissue and background pixels (6). Pixels are grouped in 4 clusters (as typically performed for attenuation correction in PET/MR imaging (7)), and the 3 clusters with the highest intensity are selected. The mean PD image is filtered with a Gaussian kernel (5 mm standard deviation) to suppress abnormally high intensity regions, and a quadratic polynomial surface is fitted to the selected pixels to estimate a smooth coil sensitivity field. The surface is used to normalize all images in each slice.

**Conversion of signal intensity to gadolinium concentration**

In dual-sequence implementations for first-pass perfusion, the signal intensity must be converted to gadolinium concentration to account for signal intensity differences between the low-resolution arterial input function (AIF) slice and the high-resolution short-axis slices, as well as improve the linearity between the signal intensity and gadolinium concentration. In this study, coil sensitivity correction and conversion of signal intensity to gadolinium concentration in the low-resolution AIF slice were also important for eliminating any systematic effects on different AIF sampling locations. Conversion was performed using the signal model for a saturation recovery-prepared single-shot spoiled gradient echo sequence, as previously described (8-10). Briefly, the process starts by converting the signal intensity in each voxel and at each dynamic, $S\left( t \right),$ to the relaxation rate $R_{1}(t)$ using the following equation:

$$S\left( t \right)=\Psi\left( \left( 1-e^{-TI\cdot R_{1}\left( t \right)} \right){a\left( t \right)}^{n-1}+\left( 1-e^{-TR\cdot R_{1}\left( t \right)} \right)\frac{1-{a\left( t \right)}^{n-1}}{1-a\left( t \right)} \right) (1)$$

where $\Psi$ is a calibration constant dependent on factors such as the receiver gain, proton density and the flip angle $\alpha$, $TI$ is the time between the saturation pulse and the central line of *k*-space, $TR$ is the repetition time (time between phase encoding steps), $n$ is the number of excitations between the start of acquisition and the central line of *k*-space, and $a\left( t \right)=e^{-TR\cdot R_{1}(t)}\cos\alpha$. $\Psi$ can be measured with equation 1 using the pre-contrast baseline intensity $S\left( 0 \right)$ and the native relaxation rate $R_{1}(0)$, obtained in T_1_ maps acquired prior to perfusion scans ($R_{1}\left( 0 \right)=1/T_{1}(0)$). In our implementation, the $S\left( 0 \right)$ in each pixel is the mean of the first 5 baseline dynamics following filtering of each image with a Gaussian kernel (1 mm standard deviation).

The estimated $R_{1}(t)$ is then inserted in the following equation to yield the gadolinium concentration in mmol/L, $C\left( t \right)$:

$$C\left( t \right)=\frac{1}{r_{1}}\left( R_{1}(t)-R_{1}(0) \right) (2)$$

where $r_{1}$ is the relaxivity of the contrast agent. The above formula assumes that $r_{1}$ does not change during the passage of contrast agent in tissue and that $R_{1}(t)$ is linearly proportional to $C\left( t \right)$.

**References**

1. Jacobs M, Benovoy M, Chang LC, Arai AE, Hsu LY. Evaluation of an automated method for arterial input function detection for first-pass myocardial perfusion cardiovascular magnetic resonance. J Cardiovasc Magn Reson 2016;18:17.

2. Otsu N. A Threshold Selection Method from Gray-Level Histograms. IEEE Transactions on Systems, Man, and Cybernetics 1979;9:62-66.

3. Nocedal J, Wright SJ. Numerical Optimization. New York: Springer-Verlag 1999.

4. Mattes D, Haynor DR, Vesselle H, Lewellyn TK, Eubank W. Nonrigid multimodality image registration. Medical Imaging 2001: Image Processing: SPIE Publications, 2001:1609–1620.

5. Hsu LY, Rhoads KL, Holly JE, Kellman P, Aletras AH, Arai AE. Quantitative myocardial perfusion analysis with a dual-bolus contrast-enhanced first-pass MRI technique in humans. J Magn Reson Imaging 2006;23:315-22.

6. Lloyd SP. Least-Squares Quantization in PCM. Ieee T Inform Theory 1982;28:129-137.

7. Martinez-Moller A, Souvatzoglou M, Delso G et al. Tissue Classification as a Potential Approach for Attenuation Correction in Whole-Body PET/MRI: Evaluation with PET/CT Data. Journal of Nuclear Medicine 2009;50:520-526.

8. Henderson E, Sykes J, Drost D, Weinmann HJ, Rutt BK, Lee TY. Simultaneous MRI measurement of blood flow, blood volume, and capillary permeability in mammary tumors using two different contrast agents. J Magn Reson Imaging 2000;12:991-1003.

9. Biglands J, Magee D, Boyle R, Larghat A, Plein S, Radjenovic A. Evaluation of the effect of myocardial segmentation errors on myocardial blood flow estimates from DCE-MRI. Phys Med Biol 2011;56:2423-43.

10. Papanastasiou G, Williams MC, Dweck MR et al. Quantitative assessment of myocardial blood flow in coronary artery disease by cardiovascular magnetic resonance: comparison of Fermi and distributed parameter modeling against invasive methods. J Cardiovasc Magn Reson 2016;18:57.

**Table S1. Pairwise comparisons for MBF and MPR**

|  | | | **LA** | **bLV** | **mLV** | **aLV** |  |
| --- | --- | --- | --- | --- | --- | --- | --- |
| **Rest MBF** | **Normal** | **bLV** | ***< 0.001*** |  |  |  | *χ*^2^(4) = 165.35, *p* < 0.001 |
|  |  | **mLV** | ***< 0.001*** | ***0.001*** |  |  |  |
|  |  | **aLV** | ***< 0.001*** | ***< 0.001*** | 1.000 |  |  |
|  |  | **AoR** | 1.000 | ***< 0.001*** | ***< 0.001*** | ***< 0.001*** |  |
|  | **Abnormal** | **bLV** | ***< 0.001*** |  |  |  | *χ*^2^(4) = 105.58, *p* < 0.001 |
|  |  | **mLV** | ***< 0.001*** | 0.397 |  |  |  |
|  |  | **aLV** | ***< 0.001*** | 1.000 | 0.980 |  |  |
|  |  | **AoR** | 1.000 | ***< 0.001*** | ***< 0.001*** | ***< 0.001*** |  |
| **Stress MBF** | **Normal** | **bLV** | 1.000 |  |  |  | *χ*^2^(4) = 68.56, *p* < 0.001 |
|  |  | **mLV** | **0.014** | 0.984 |  |  |  |
|  |  | **aLV** | ***< 0.001*** | **0.016** | 1.000 |  |  |
|  |  | **AoR** | **0.042** | ***< 0.001*** | ***< 0.001*** | ***< 0.001*** |  |
|  | **Abnormal** | **bLV** | ***< 0.001*** |  |  |  | *χ*^2^(4) = 136.35, *p* < 0.001 |
|  |  | **mLV** | ***< 0.001*** | 0.200 |  |  |  |
|  |  | **aLV** | ***0.001*** | 0.253 | ***< 0.001*** |  |  |
|  |  | **AoR** | 1.000 | ***< 0.001*** | ***< 0.001*** | ***< 0.001*** |  |
| **MPR** | **Normal** | **bLV** | 1.000 |  |  |  | *χ*^2^(4) = 31.20, *p* < 0.001 |
|  |  | **mLV** | ***< 0.001*** | ***0.005*** |  |  |  |
|  |  | **aLV** | ***0.001*** | 0.114 | 1.000 |  |  |
|  |  | **AoR** | **0.049** | 1.000 | 0.321 | 1.000 |  |
|  | **Abnormal** | **bLV** | 1.000 |  |  |  | *χ*^2^(4) = 93.24, *p* < 0.001 |
|  |  | **mLV** | ***< 0.001*** | ***< 0.001*** |  |  |  |
|  |  | **aLV** | ***< 0.001*** | ***< 0.001*** | ***< 0.001*** |  |  |
|  |  | **AoR** | 1.000 | 0.226 | ***< 0.001*** | **0.032** |  |

Values in bold are significant at the 0.05 level and values in bold-italic are significant at the 0.01 level. Overall significance is reported on the right with the following format: *χ*^2^(degrees of freedom) = chi-square metric, *p*-value. aLV: apical left ventricle; AoR: aortic root; bLV: basal left ventricle; LA: left atrium; MBF: myocardial blood flow; mLV: mid left ventricle; MPR: myocardial perfusion reserve.

**Table S2. Pairwise comparisons for ROC curve analysis**

|  |  | **LA** | **bLV** | **mLV** | **aLV** |
| --- | --- | --- | --- | --- | --- |
| **Stress MBF** | **bLV** | 1.000 |  |  |  |
|  | **mLV** | ***< 0.001*** | ***< 0.001*** |  |  |
|  | **aLV** | 0.647 | 1.000 | **0.027** |  |
|  | **AoR** | ***< 0.001*** | ***< 0.001*** | ***< 0.001*** | ***< 0.001*** |
| **MPR** | **bLV** | 1.000 |  |  |  |
|  | **mLV** | ***< 0.001*** | ***< 0.001*** |  |  |
|  | **aLV** | 1.000 | 1.000 | ***< 0.001*** |  |
|  | **AoR** | 1.000 | 0.717 | ***< 0.001*** | 0.795 |

Values in bold are significant at the 0.05 level and values in bold-italic are significant at the 0.01 level. aLV: apical left ventricle; AoR: aortic root; bLV: basal left ventricle; LA: left atrium; MBF: myocardial blood flow; mLV: mid left ventricle; MPR: myocardial perfusion reserve.

**Table S3. Pairwise comparisons for stress MBF versus MPR ROC curves**

|  | | **Stress MBF** | | | | |
| --- | --- | --- | --- | --- | --- | --- |
|  |  | **LA** | **bLV** | **mLV** | **aLV** | **AoR** |
| **MPR** | **LA** | 1.000 | 1.000 | ***0.007**** | 1.000 | **0.049** |
|  | **bLV** | 1.000 | 1.000 | 0.064 | 1.000 | ***0.008*** |
|  | **mLV** | ***< 0.001*** | ***< 0.001*** | 1.000 | 0.082 | ***< 0.001*** |
|  | **aLV** | 1.000 | 1.000 | 0.106 | 1.000 | ***< 0.001*** |
|  | **AoR** | 1.000 | 1.000 | ***0.004**** | 1.000 | 0.086 |

Values in bold are significant at the 0.05 level and values in bold-italic are significant at the 0.01 level. Statistically significant p-values favor stress MBF except for those with an asterisk (*) that favor MPR. aLV: apical left ventricle; AoR: aortic root; bLV: basal left ventricle; LA: left atrium; MBF: myocardial blood flow; mLV: mid left ventricle; MPR: myocardial perfusion reserve.
